# Supplementary material for: Melatonin Ameliorates Cadmium Toxicity in Tobacco Seedlings by Depriving Its Bioaccumulation, Enhancing Photosynthetic Activity and Antioxidant Gene Expression
Source: Plants (Basel). 2024 Oct 31;13(21):3049. doi: 10.3390/plants13213049 (PMC11548336; doi:10.3390/plants13213049)
Supplement: Supplementary file 1 [file plants-13-03049-s001.zip › plants-3269118-supplementary.pdf]

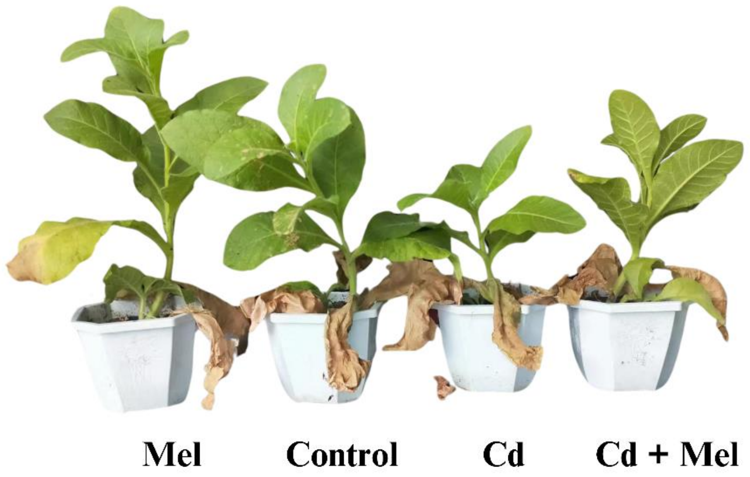

Figure S1. Growth responses of tobacco seedlings under different treatments. Cd, cadmium; Mel, melatonin application.
